# Preventing Failures by Dataset Shift Detection in Safety-Critical Graph Applications

**DOI:** 10.3389/frai.2021.589632

**Published:** 2021-05-18

**Authors:** Hoseung Song, Jayaraman J. Thiagarajan, Bhavya Kailkhura

**Affiliations:** ^1^ Department of Statistics, University of California, Davis, CA, United States; ^2^ Lawrence Livermore National Laboratory, Livermore, CA, United States

**Keywords:** graph learning, dataset shift, safety, two-sample testing, random graph models

## Abstract

Dataset shift refers to the problem where the input data distribution may change over time (e.g., between training and test stages). Since this can be a critical bottleneck in several safety-critical applications such as healthcare, drug-discovery, etc., dataset shift detection has become an important research issue in machine learning. Though several existing efforts have focused on image/video data, applications with graph-structured data have not received sufficient attention. Therefore, in this paper, we investigate the problem of detecting shifts in graph structured data through the lens of statistical hypothesis testing. Specifically, we propose a practical two-sample test based approach for shift detection in large-scale graph structured data. Our approach is very flexible in that it is suitable for both undirected and directed graphs, and eliminates the need for equal sample sizes. Using empirical studies, we demonstrate the effectiveness of the proposed test in detecting dataset shifts. We also corroborate these findings using real-world datasets, characterized by directed graphs and a large number of nodes.

## 1 Introduction

Most machine learning (ML) applications, e.g., healthcare, drug-discovery, etc., encounter dataset shift when operating in the real-world. The reason for this comes from the bias in the testing conditions compared to the training environment introduced by experimental design. It is well known that ML systems are highly susceptible to such dataset shifts, which often leads to unintended and potentially harmful behavior. For example, in ML-based electronic health record systems, input data is often characterized by shifting demographics, where clinical and operational practices evolve over time and a wrong prediction can threaten human safety.

Although dataset shift is a frequent cause of failure of ML systems, very few ML systems inspect incoming data for a potential distribution shift (Bulusu et al., 2020). While some practical methods such as (Rabanser et al., 2019) have been proposed for detecting shifts in applications with Euclidean structured data (speech, images, or video), there are limited efforts in solving such issues for graph structured data that naturally arises in several scientific and engineering applications. In recent years there has been a surge of interest in applying ML techniques to structured data, e.g. graphs, trees, manifolds etc. In particular, graph structured data is becoming prevalent in several high-impact applications including bioinformatics, neuroscience, healthcare, molecular chemistry and computer graphics. In this paper, we investigate the problem of detecting distribution shifts in graph-structured datasets for responsible deployment of ML in safety-critical applications. Specifically, we propose to solve the problem of detecting shifts in graph-structured data through the lens of statistical two-sample testing. Broadly, the objective in two-sample testing for graphs is to test whether two populations of random graphs are different or not based on the samples generated from each of them.

Two-sample testing has been of significant research interest due to its broad applicability. An important class of testing methods relies on summary metrics that quantify the topological differences between networks. For example, in brain network analysis, commonly adopted topological summary metrics include the global efficiency (Ginestet et al., 2011) and network modularity (Ginestet et al., 2014). An inherent challenge with these approaches is that the topological characteristics depend directly on the number of edges in the graph, and can be insufficient in practice. An alternative class of methods is based on comparing the structure of subgraphs to produce a similarity score (Shervashidze et al., 2009; Macindoe and Richards, 2010). For example, Shervashidze et al. (2009) used the earth mover’s distance between the distributions of feature summaries of their constituent subgraphs.

While these heuristic methods are reasonably effective for comparing real-world graphs, not until recently that a principled analysis of hypothesis testing with random graphs was carried out. In this spirit, Ginestet et al. (2017) developed a test statistic based on a precise geometric characterization of the space of graph Laplacian matrices. Most of these approaches for graph testing based on classical two-sample tests are only applicable to the restrictive low-dimensional setting, where the population size (number of graphs) is larger than the size of the graphs (number of vertices). To overcome this challenge, Tang et al. (2017a) proposed a semi-parametric two-sample test for a class of latent position random graphs, and studied the problem of testing whether two dot product random graphs are drawn from the same population or not. Other testing approaches that focused on hypothesis testing for specific scenarios, such as sparse networks (Ghoshdastidar et al., 2017a) and networks with a large number of nodes (Ghoshdastidar et al., 2017b), have been developed. More recently, Ghoshdastidar and von Luxburg (2018) developed a novel testing framework for random graphs, particularly for the cases with small sample sizes and the large number of nodes, and studied its optimality. More specifically, this test statistic was based on the asymptotic null distributions under certain model assumptions.

Unfortunately, all these approaches are limited to testing undirected graphs under the equal sample size (for two graph populations) setting. In real-world dataset shift detection problems, these assumptions are extremely restrictive, making existing approaches inapplicable to several applications. In order to circumvent these crucial shortcomings, we develop a novel approach based on hypothesis testing for detecting shifts in graph-structured data, which is more flexible (i.e., accommodates 1) both undirected and directed graphs and 2) unequal sample size cases). Moreover, it is highly effective even when the sample size grows. Notice that, similar to the setting in Ghoshdastidar and von Luxburg (2018), we also consider scenarios where all networks are defined from the same vertex set, which is common to several real-world applications. The main contributions of this paper are summarized below:

• We propose a new test statistic that can be applied to undirected graphs as well as directed graphs and/or unweighted graphs as well as weighted graphs, while eliminating the equal sample size requirement. The asymptotic distribution for the proposed statistic, based on the well-known U-statistic, is derived.
• A practical permutation approach based on a simplified form of the statistic is also proposed.
• We compare the new approach with existing methods for graph testing in diverse simulation settings, and show that the proposed statistic is more flexible and achieves significant performance improvements.
• In order to demonstrate the usefulness of the proposed method in challenging real-world problems, we consider several applications (including a healthcare application), and show the effectiveness of our approach.

## 2 Preliminaries

We consider the following two-sample setting. Let two random graph populations with *d* vertices be denoted as $A_{1},\ldots ,A_{m}$ from $P\in {\left[0,1\right]}^{d\times d}$ and ${ℬ}_{1},\ldots ,{ℬ}_{n}$ from $Q\in {\left[0,1\right]}^{d\times d}$ with their adjacency matrices $A_{1},\ldots ,A_{m}$ and $B_{1},\ldots ,B_{n}$, respectively. We are concerned with testing hypotheses:

$$H_{0}:P=Q\;\text{ vs}\;\;H_{1}:P\neq Q.$$ (1)

Notice that we consider the cases where each population consists of independent and identically distributed samples, which encompasses a wide-range of network analysis problems, see, e.g., Holland et al. (1983), Newman and Girvan (2004), Newman (2006). In contrast to existing formulations, e.g., Ghoshdastidar and von Luxburg (2018), we consider a more flexible setup where 1) the sample sizes *m* and *n* are allowed to be different and 2) the graphs in *p* and *Q* can be weighted and/or directed.

While there have several efforts to two-sample testing of graphs (Bubeck et al., 2016; Gao and Lafferty, 2017; Maugis et al., 2017), recent works such as Tang et al. (2017a), Tang et al. (2017b); Ginestet et al. (2017) have focused on designing more general testing methods that are applicable to practical settings. For example, Ginestet et al. (2017) proposed a practical test statistic based on the correspondence between an undirected graph and its Laplacian under the inhomogeneous Erdős-Rényi (IER) assumption, which means all nodes are independently generated from a Bernoulli distribution (see details in Section 3). The test statistic, under the assumption of equal sample sizes *m*, can be described as follows:

$$T_{gin}=\sum_{i<j}^{d}\frac{{\left[{\left(\bar{A}\right)}_{ij}-{\left(\bar{B}\right)}_{ij}\right]}^{2}}{a},$$ (2)

where

$$a=\frac{1}{m\left(m-1\right)}\sum_{k=1}^{m}{\left[{\left(A_{k}\right)}_{ij}-{\left(\bar{A}\right)}_{ij}\right]}^{2}+\frac{1}{m\left(m-1\right)}\sum_{k=1}^{m}{\left[{\left(B_{k}\right)}_{ij}-{\left(\bar{B}\right)}_{ij}\right]}^{2},$$

$${\left(\bar{A}\right)}_{ij}=\frac{1}{m}\sum_{k=1}^{m}{\left(A_{k}\right)}_{ij},\;\;{\left(\bar{B}\right)}_{ij}=\frac{1}{m}\sum_{k=1}^{m}{\left(B_{k}\right)}_{ij}.$$

The authors showed that $T_{gin}$ converges to a chi-square distribution as m→∞ under $H_{0}$. However, this statistic can be interpreted as Hotelling’s $T^{2}$ statistic for multivariate data, thus leading to no performance guarantees for “small *m* and large *d*” scenario. This is because the variance estimates used in Eq. 2 are not stable for small *m* and large *d*, especially when graphs are sparse.

Recently, Ghoshdastidar and von Luxburg (2018) proposed a new class of test statistics, designed for different scenarios under the IER model assumption. More specifically, they focused on cases with small *m* and large *d*. For cases with m>1, the following test statistic was used:

$$T_{spec}=\frac{{\left|\left|\sum_{k=1}^{m}\left(A_{k}-B_{k}\right)\right|\right|}_{2}}{\sqrt{\underset{1\leq i\leq d}{\max}\sum_{j=1}^{d}\sum_{j=1}^{d}\sum_{k=1}^{m}\left[{\left(A_{k}\right)}_{ij}+{\left(B_{k}\right)}_{ij}\right]}},$$ (3)

While it was suggested by the authors to perform this test using bootstraps from the aggregated data, this could be challenging for sparse graphs, since it is difficult to construct bootstrapped statistics from an operator norm. Hence, they considered an alternate test statistic based on the Frobenius-norm as follows:

$$T_{fro}=\frac{\sum_{i<j}^{d}\left(\sum_{k\leq m/2}{\left({\text{Δ}}_{k}\right)}_{ij}\right)\left(\sum_{k>m/2}{\left({\text{Δ}}_{k}\right)}_{ij}\right)}{\sqrt{\sum_{i<j}^{d}\left(\sum_{k\leq m/2}{\left(S_{k}\right)}_{ij}\right)\left(\sum_{k>m/2}{\left(S_{k}\right)}_{ij}\right)}},$$ (4)

where ${\left({\text{Δ}}_{k}\right)}_{ij}={\left(A_{k}\right)}_{ij}-{\left(B_{k}\right)}_{ij}$ and ${\left(S_{k}\right)}_{ij}={\left(A_{k}\right)}_{ij}+{\left(B_{k}\right)}_{ij}$. It was shown that this test is provably effective and more reliable. Furthermore, they derived the asymptotic normality of $T_{fro}$ as d→∞ to make the method instantly applicable without the bootstrap procedure. Despite the good properties of this method, this test can be used only when the two sample sizes are equal, and when graphs are undirected. In the rest of this paper, we develop a new test statistic which addresses these two crucial limitations.

## 3 Proposed Test

To carry out two-sample testing, we want to measure the distance between two populations. Here, we utilize the Frobenius distance as the evidence for discrepancy between two populations:

$$T={\left\|P-Q\right\|}_{F}^{2}.$$ (5)

Next, we provide finite sample estimates of this quantity. To accommodate more general settings for random graphs, the new test statistic is defined as follows:

$$T_{new}=\overset{d}{\underset{i=1}{\sum }}\overset{d}{\underset{j=1}{\sum }}T_{ij},$$ (6)

where

$$T_{ij}=\frac{1}{m\left(m-1\right)}\overset{m}{\underset{k\neq l}{\sum }}{\left(A_{k}\right)}_{ij}{\left(A_{l}\right)}_{ij}+\frac{1}{n\left(n-1\right)}\overset{n}{\underset{k\neq l}{\sum }}{\left(B_{k}\right)}_{ij}{\left(B_{l}\right)}_{ij}-\frac{2}{mn}\overset{m}{\underset{k=1}{\sum }}\overset{n}{\underset{l=1}{\sum }}{\left(A_{k}\right)}_{ij}{\left(B_{l}\right)}_{ij}.$$

Note that the proposed test statistic accommodates scenarios where 1) the sample sizes *m* and *n* are different and 2) the graphs in *p* and *Q* are weighted and/or directed.

Next, we analyze the theoretical properties of the proposed test. For the ease of theoretical analysis, we focus on the case where graphs are unweighted and undirected. However, the proposed test and algorithmic tools are applicable to weighted and/or directed graph scenarios which is the main focus of the paper and is considered in our experimental evaluations. More specifically, in our theoretical analysis, we assume that graphs are drawn from the inhomogeneous Erdős-Rényi (IER) random graph process, which is considered as an extended version of the Erdős-Rényi (ER) model from Bollobás et al. (2007). In other words, we consider unweighted and undirected random graphs, where edges occur independently without any additional structural assumption on the population adjacency matrix. Note, the IER model encompasses other models studied in the literature including random dot product graphs (Tang et al., 2017b) and stochastic block models (Lei et al., 2016). A graph $G\in {\left[0,1\right]}^{d\times d}$ from a population symmetric adjacency *p* with zero diagonal is considered to be an IER graph if ${\left(G\right)}_{ij}\overset{i.i.d}{}Bernoulli\left(P_{ij}\right)$ for all i,j∈{1,…,d}. Here, *d* denotes the cardinality of the vertex set. Next we analyze the theoretical properties of the proposed test under IER assumption.

LEMMA 3.1. $T_{new}$ is an unbiased empirical estimate of T, that is,

$$E\left(T_{new}\right)=T.$$ (7)

PROOF. Under the IER assumptions, for all i,j=1,…,d, we have

$${\left(A_{k}\right)}_{ij}{\left(A_{l}\right)}_{ij}∼Bernoulli\left(P_{ij}^{2}\right),$$

$$\sum_{k\neq l}^{m}{\left(A_{k}\right)}_{ij}{\left(A_{l}\right)}_{ij}∼Binomial\left(m\left(m-1\right),P_{ij}^{2}\right),$$

$${\left(B_{k}\right)}_{ij}{\left(B_{l}\right)}_{ij}∼Bernoulli\left(Q_{ij}^{2}\right),$$

$$\overset{n}{\underset{k\neq l}{\sum }}{\left(B_{k}\right)}_{ij}{\left(B_{l}\right)}_{ij}∼Binomial\left(n\left(n-1\right),Q_{ij}^{2}\right),$$

since $A_{k}$ and $B_{l}$ are mutually independent (k=1,…,m,   l=1,…,n). Then,

$$\begin{array}{c} E\left(T\right)=\sum_{i=1}^{d}\sum_{j=1}^{d}\left[\frac{1}{m\left(m-1\right)}m\left(m-1\right)P_{ij}^{2}+\frac{1}{n\left(n-1\right)}n\left(n-1\right)Q_{ij}^{2}-\frac{2}{mn}mnP_{ij}Q_{ij}\right]\\ =\sum_{i=1}^{d}\sum_{j=1}^{d}{\left(P_{ij}-Q_{ij}\right)}^{2}=∥P-Q{∥}_{F}^{2}. \end{array}$$

In the form of $T_{ij}$, the first term and the second term represent a similarity (closeness) within two samples, and the last term represents similarity between two samples. Hence, a relatively large value of $T_{new}$ is the evidence against the null hypothesis. Note that the proposed statistic does not require equal sample sizes and undirected graphs assumptions.

When m=n, we have a simpler form of the estimate. Let $Z=\left(z_{1},\ldots ,z_{m}\right)$ be i.i.d random variables $z_{k}=\left(A_{k},B_{k}\right)∼P\times Q\;\;\left(k=1,\ldots ,m\right)$. Then,

$$T_{new}=\sum_{i=1}^{d}\sum_{j=1}^{d}T_{ij},$$ (8)

where

$$T_{ij}=\frac{1}{m\left(m-1\right)}\sum_{k\neq l}^{m}h{\left(u_{k},u_{l}\right)}_{ij},$$ (9)

and $h{\left(z_{k},z_{l}\right)}_{ij}={\left(A_{k}\right)}_{ij}{\left(A_{l}\right)}_{ij}+{\left(B_{k}\right)}_{ij}{\left(B_{l}\right)}_{ij}-{\left(A_{k}\right)}_{ij}{\left(B_{l}\right)}_{ij}-{\left(A_{l}\right)}_{ij}{\left(B_{k}\right)}_{ij}$. Since the proposed estimate has a form of *U*-statistics, which provides a minimum-variance unbiased estimator for *T* (Hoeffding, 1992; Serfling, 2009), the asymptotic distribution of $T_{new}$ can be derived based on the asymptotic results of *U*-statistics.

Theorem 3.1 Assume $E\left(h^{2}\right)<\infty$. Under $H_{1}$, we have

$$\sqrt{m}\left(T_{new}-T\right)\overset{d}{\to }N\left(0,d^{2}\sigma^{2}\right),$$ (10)

where $\sigma^{2}={\mathrm{var}}_{z}\left(E_{z'}h{\left(z,z^{\prime }\right)}_{ij}\right)$. Under $H_{0}$, the U-statistic is degenerate and

$$mT_{new}\overset{d}{\to }\sum_{u=1}^{\infty }d^{2}\lambda_{u}\left(\xi_{u}^{2}-1\right),$$ (11)

where $\xi_{u}\overset{i.i.d}{}N\left(0,1\right)$ and $\lambda_{u}$ are the solutions of

$$\lambda_{u}\phi_{u}\left(z\right)=\int_{z^{\prime }}h{\left(z,z^{\prime }\right)}_{ij}\phi_{u}\left(z^{\prime }\right)dP\left(z^{\prime }\right).$$ (12)

PROOF. These results can be obtained by applying the asymptotic properties of *U*-statistics as given in Serfling (2009) and the IER assumptions.

Having devised the test statistic, our next aim is to determine whether the new test statistic $T_{new}$ is large enough to be outside the 1−α quantile of the limiting null distribution in Eq. 11, where *a* is the significance level of the test. One difficulty in implementing this test is that the asymptotic null distribution 11) and its *a* quantile do not have an analytic form unless $\lambda_{u}=0$ or 1. Therefore, in order to estimate this quantile, we propose a permutation approach on the aggregated data. The main advantage of this method is that it yields a valid level *a* test in finite-sample scenarios (Lehmann and Romano, 2006). To this end, we first consider a simpler form of the test statistic (based on $T_{new}$) defined as follows:

$${T^{\prime }}_{new}=\sum_{i=1}^{d}\sum_{j=1}^{d}{T^{\prime }}_{ij},$$ (13)

where

$${T^{\prime }}_{ij}=\frac{1}{m\left(m-1\right)}\sum_{k\neq l}^{m}{\left(A_{k}\right)}_{ij}{\left(A_{l}\right)}_{ij}+\frac{1}{n\left(n-1\right)}\sum_{k\neq l}^{n}{\left(B_{k}\right)}_{ij}{\left(B_{l}\right)}_{ij}.$$ (14)

Although we do not use the last term of $T_{ij}$ in the definition of ${T^{\prime }}_{ij}$, the performance of the test statistic ${T^{\prime }}_{new}$ achieved by incorporating similarities in two samples is still maintained in the permutation framework. The permutation test is summarized in **Algorithm 1**; its computational cost is $O\left(R{\left(m\vee n\right)}^{2}\right)$, where (m∨n) indicates the maximum among *m* and *n*.

Algorithm 1 Permutation test using ${T^{\prime }}_{new}$.

**Input:** Graph samples $A_{1},\ldots ,A_{m}$ and ${ℬ}_{1},\ldots ,{ℬ}_{n}$; Significance level α; Number of permutation *R*.
**Output:** Reject the null hypothesis $H_{0}$ if *p*-value ≤α.
1: Compute $T{'}_{new}$ by Eqs. 13, 14.
2: **for** r=1 to *R* **do**
3: Randomly permute the pooled samples $\left\{A_{1},\ldots ,A_{m},\;{ℬ}_{1},\ldots ,{ℬ}_{n}\right\}$ and divide into two groups with sample sizes m and n.
4: Compute ${T^{\prime }}_{r}$ which is ${T^{\prime }}_{new}$ (as given in Eqs. 13, 14 calculated using permuted samples.
5: **end for**.
6: Calculate *p*-value = $\left|\left\{r:{T^{\prime }}_{r}\geq {T^{\prime }}_{new}\right\}/R\right|$

Unlike Ghoshdastidar and von Luxburg (2018) where the test is reliable even for a small number of samples, due to its asymptotic distribution, our test procedure needs a reasonable number of samples to implement the permutation test. Based on simulations, we see that as low as four samples are sufficient to obtain reliable results.

## 4 Experiments

Here, we first examine the performance of the new test statistics under diverse settings through simulation studies. Later, we will apply the new test to real-world applications.

### 4.1 Simulated Data

To evaluate the performance of the new test, we examine sparse graphs from stochastic block models with two communities as studied in Tang et al. (2017a) an Ghoshdastidar and von Luxburg (2018). Specifically, we consider sparse graphs with *d* nodes where the same d/2 size community is constructed with an edge probability *p* and d/2 size different community with an edge probability *q*. In other words, we define *p* and *Q* as follows:

$$P:{\left(\begin{array}{cc} p & q\\ q & p \end{array}\right)}_{d\times d}\;\;vs\;\;Q:{\left(\begin{array}{cc} p+\epsilon & q\\ q & p+\epsilon \end{array}\right)}_{d\times d}.$$

We generate *m* samples from *p* and *n* samples from *Q*. Under the null, ϵ=0, implying P=Q, whereas ϵ>0 under $H_{1}$, implying P≠Q. Following Ghoshdastidar and von Luxburg (2018), we set p=0.1, q=0.05, and ϵ=0 for null, whereas ϵ=0.04 for the alternative hypothesis. We examine the performance of the new test for different choices of d∈{100,200,300,400,500}.

The performance of the test based on ${T^{\prime }}_{new}$ is studied and compared to existing methods. T_fro in Ghoshdastidar and von Luxburg (2018) is the bootstrap test based on $T_{fro}$, and T_asymp denotes the normal dominance test based on the asymptotic distribution of $T_{fro}$ (also from Ghoshdastidar and von Luxburg (2018)). We denote the new test which is the permutation test based on ${T^{\prime }}_{new}$ as $T^{\prime }\_new$. The estimated power is calculated as the number of null rejections at α=0.05 level out of 100 independent trials for each of these methods. For T_fro and T_new, *p*-values are determined by 1,000 permutation runs to have a reliable comparison.

**FIGURE 1:**
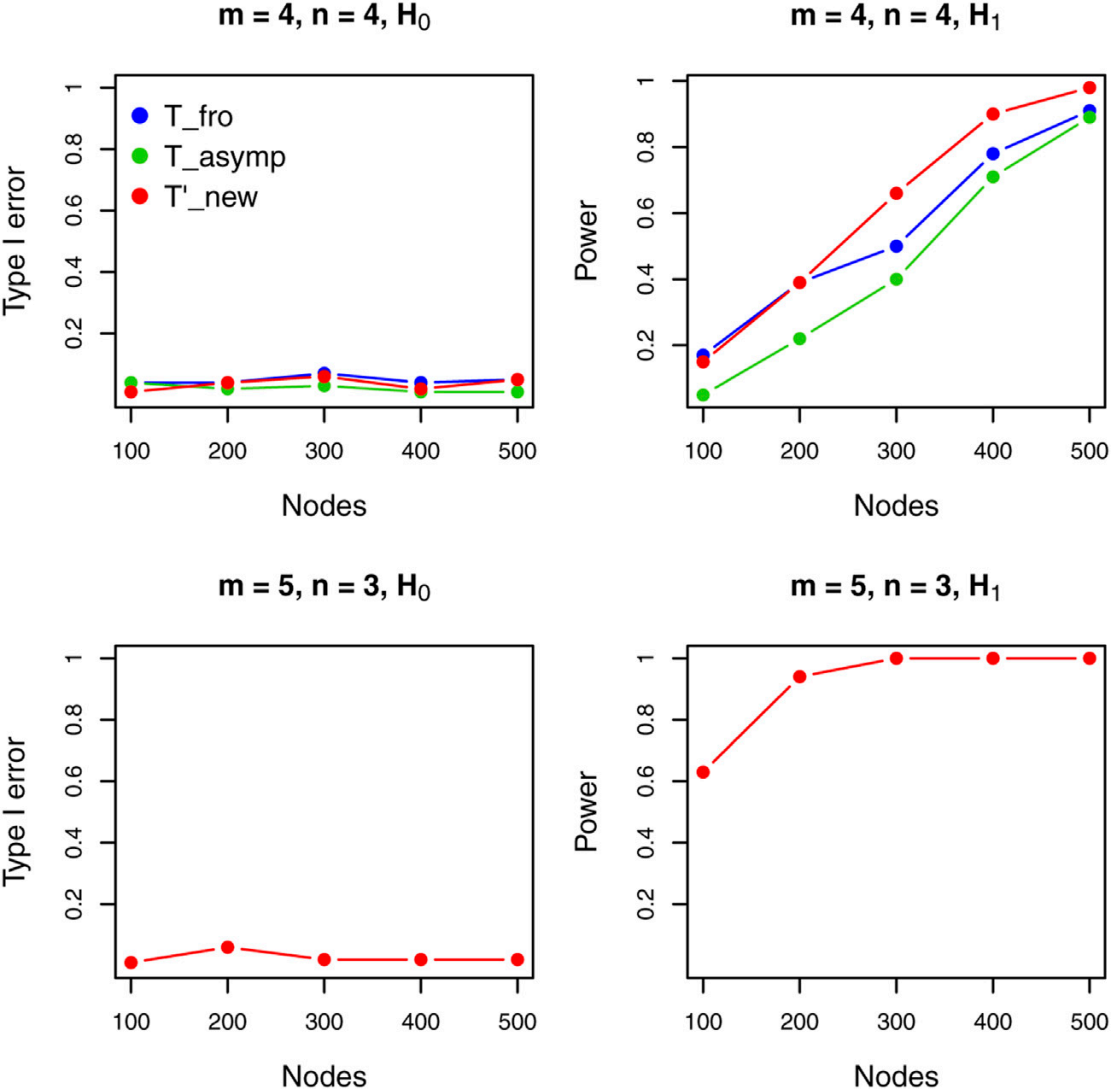
Performance comparison of different tests for undirected graphs.

Figure 1 shows results for the undirected graph case under different settings. When two sample sizes are equal (upper panels), where existing methods can be applied, we see that the proposed test outperforms all other methods. Note that, when the sample size of two graph populations are different (i.e., m≠n), the existing methods cannot be applied. We see that the proposed test still performs well under sample imbalance and the large *d* regime.

**FIGURE 2:**
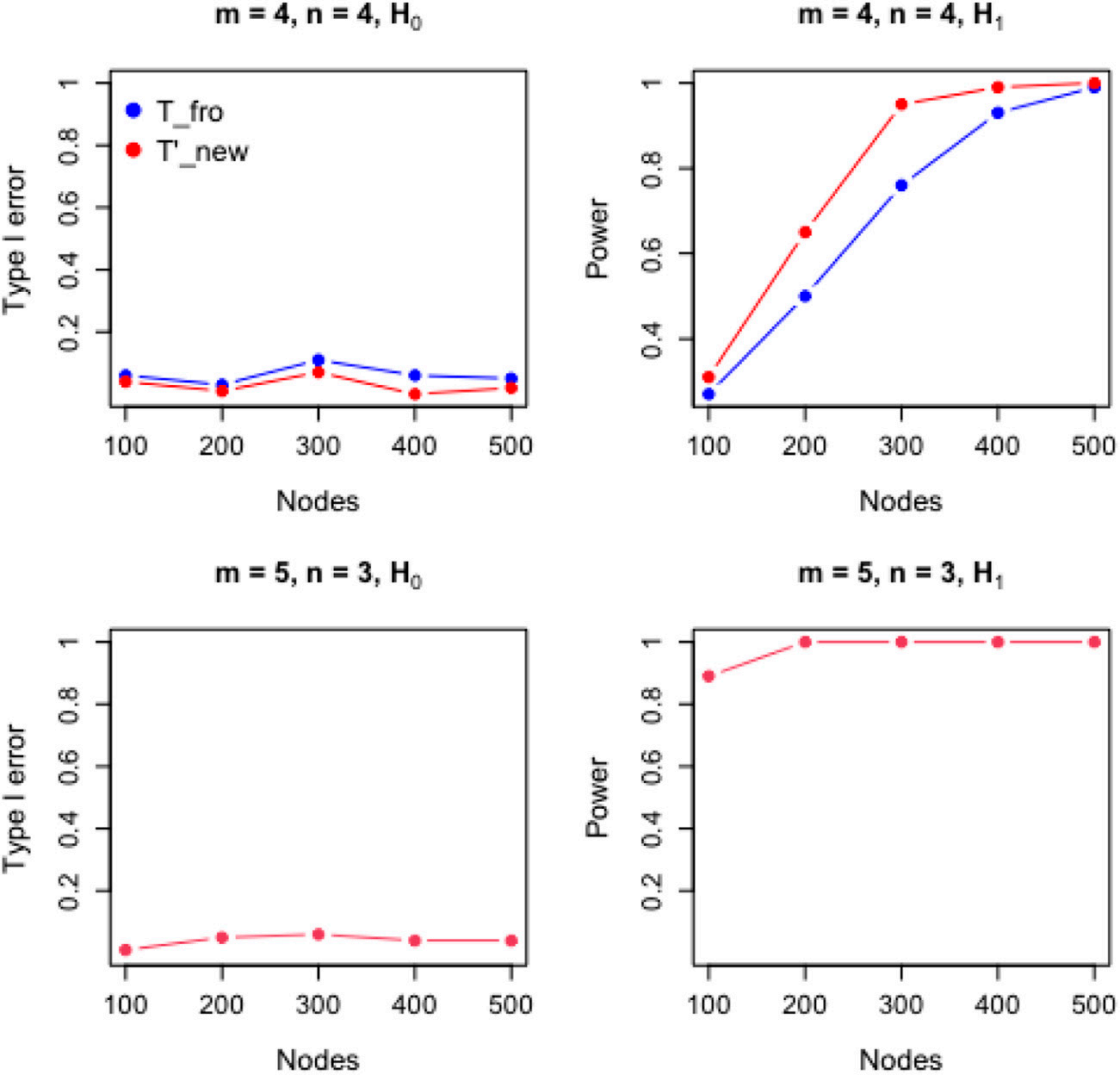
Performance comparison of proposed test for directed graphs.

We also evaluate the performance of the new test for directed graphs under various configurations. (Figure 2). The existing methods are not applicable to directed graphs, but we transform $T_{fro}$ so that it can be applied to directed graphs. The results show that the new test also has better power than the existing method in two-sample testing for directed graph and works well for large graphs.

Next, we examine the effect of the sparsity on the performance of the tests. To this end, we consider the same setting as above, but with different choices of ϵ∈{0.02,0.03,0.04} for each of methods. Small ϵ implies that there is small difference between *p* and *Q*, making the tests more difficult to detect discrepancy between two samples. Table 1 shows results for undirected graphs with variations in the sparsity level ϵ. We see that, in general, the proposed method is consistently superior to existing methods. This indicates that our test statistic is more effective in detecting the inhomogeneity between two samples than the existing methods. The effect of a sparsity level ϵ on the performance of the proposed test for directed graphs can be found in Table 2. We see that the proposed test also performs better than the existing method for directed graph settings, and as expected, the power increases as ϵ or the number of samples increases.

**TABLE 1:** Power comparison of different tests for undirected graphs with varying sparsity levels.

m=n=4	ϵ=0.02			ϵ=0.03			ϵ=0.04		
*D*	T_fro	T_asymp	$T^{\prime }\_new$	T_fro	T_asymp	$T^{\prime }\_new$	T_fro	T_asymp	$T^{\prime }\_new$
100	0.09	0.05	**0.10**	**0.10**	0.03	0.08	**0.17**	0.05	**0.17**
200	**0.09**	0.05	0.07	**0.18**	0.10	**0.18**	**0.39**	0.22	**0.39**
300	**0.17**	0.03	**0.17**	0.34	0.19	**0.37**	0.50	0.40	**0.66**
400	0.11	0.09	**0.15**	0.40	0.26	**0.53**	0.78	0.71	**0.90**
500	**0.22**	0.08	**0.22**	0.63	0.48	**0.75**	0.91	0.89	**0.98**

m=n=8	ϵ=0.02			ϵ=0.03			ϵ=0.04		
*d*	T_fro	T_asymp	$T^{\prime }\_new$	T_fro	T_asymp	$T^{\prime }\_new$	T_fro	T_asymp	$T^{\prime }\_new$
100	**0.13**	0.05	0.08	0.17	0.08	**0.23**	0.39	0.21	**0.64**
200	0.19	0.09	**0.31**	0.40	0.20	**0.67**	0.80	0.66	**0.99**
300	0.36	0.22	**0.49**	0.73	0.58	**0.92**	0.98	0.94	**1.00**
400	0.37	0.19	**0.61**	0.92	0.86	**1.00**	**1.00**	0.99	**1.00**
500	0.51	0.31	**0.76**	0.98	0.96	**1.00**	**1.00**	**1.00**	**1.00**

Bold values indicate the largest power of the test under each condition.

**TABLE 2:** Power of the proposed test for directed graphs with varying sparsity levels.

m=n=4	ϵ=0.02		ϵ=0.03		ϵ=0.04	
*D*	T_fro	$T^{\prime }\_new$	T_fro	$T^{\prime }\_new$	T_fro	$T^{\prime }\_new$
100	**0.13**	0.09	**0.11**	**0.11**	0.21	**0.26**
200	0.11	**0.12**	0.25	**0.27**	0.49	**0.66**
300	0.17	**0.22**	0.46	**0.61**	0.76	**0.94**
400	**0.20**	**0.20**	0.60	**0.72**	0.95	**1.00**
500	0.36	**0.37**	0.77	**0.93**	**1.00**	**1.00**

m=n=8	ϵ=0.02		ϵ=0.03		ϵ=0.04	
*D*	T_fro	T′_new	T_fro	T′_new	T_fro	T′_new
100	0.14	**0.18**	0.20	**0.42**	0.66	**0.93**
200	0.26	**0.38**	0.77	**0.94**	0.97	**1.00**
300	0.43	**0.68**	0.94	**1.00**	**1.00**	**1.00**
400	0.62	**0.89**	**1.00**	**1.00**	**1.00**	**1.00**
500	0.80	**0.96**	**1.00**	**1.00**	**1.00**	**1.00**

Bold values indicate the largest power of the test under each condition.

This observation becomes particularly evident when we have a large number of samples. To this end, we study how the performance of the tests is affected by the number of samples. For this study, we consider m=n∈{10,20,50} with relatively small graphs d∈{50,100,150,200} and fix ϵ=0.02. This analysis is designed to reveal the potential impact of sample size in high-dimensional settings. Tables 3, 4 report numerical results for the performance of the tests with varying number of samples. We see that the proposed test in general outperforms the existing tests for both undirected and directed graphs. Hence, we can claim that the new test works well in high-dimensional settings.

**TABLE 3:** Power comparison of different tests for undirected graphs with varying sample sizes.

	m=n=10			m=n=20			m=n=50		
*d*	T_fro	T_asymp	$T^{\prime }\_new$	T_fro	T_asymp	$T^{\prime }\_new$	T_fro	T_asymp	$T^{\prime }\_new$
50	0.08	0.08	**0.12**	0.11	0.04	**0.16**	0.28	0.15	**0.43**
100	0.16	0.08	**0.17**	0.18	0.05	**0.23**	0.61	0.42	**0.81**
150	**0.16**	0.03	0.15	0.21	0.14	**0.30**	0.70	0.52	**0.97**
200	0.14	0.06	**0.22**	0.37	0.21	**0.56**	0.94	0.89	**1.00**

Bold values indicate the largest power of the test under each condition.

**TABLE 4:** Power comparison of different tests for directed graphs with varying sample sizes.

Directed	m=n=10		m=n=20		m=n=50	
*d*	T_fro	$T^{\prime }\_new$	T_fro	$T^{\prime }\_new$	T_fro	$T^{\prime }\_new$
50	0.05	**0.09**	0.12	**0.28**	0.49	**0.77**
100	0.15	**0.24**	0.29	**0.43**	0.82	**0.99**
150	0.15	**0.21**	0.39	**0.52**	0.95	**1.00**
200	0.28	**0.42**	0.66	**0.86**	**1.00**	**1.00**

Bold values indicate the largest power of the test under each condition.

### 4.2 Real-World Applications

#### 4.2.1 Phone-Call Network

The MIT Media Laboratory conducted a study following 87 subjects who used mobile phones with a pre-installed device that can record call logs. The study lasted for 330°days from July 2004 to June 2005 (Eagle et al., 2009). Given the richness of this dataset, one question of interest to answer is that whether the phone call patterns among subjects are different between weekends and weekdays. These patterns can be viewed as a representation of the personal relationship and professional relationships of a subject. Removing days with no calls among subjects, there are t=299 networks in total (corresponding to number of days) and 87 subjects (or nodes) with adjacency matrices $N_{t}$ with value one for element (i,j) if subject *i* called *j* on day *t* and 0 otherwise. This in turn comprises of 85°days in weekends and 214°days in weekdays. This is an example of unweighted directed graphs with imbalanced sample sizes.

The test statistic and corresponding *p*-value are shown in Table 5. We see that the new test rejects the null hypothesis of equal distribution at 0.05 significance level. This outcome is intuitively plausible as phone call patterns in weekends (personal) can be different from the patterns in weekdays (work).

**TABLE 5:** Test summary on the phone-call network.

Test statistic	*p*-value
15.8131	<0.001

#### 4.2.2 Safety-Critical Healthcare Application

Modeling relationships between functional or structural regions in the brain is a significant step toward understanding, diagnosing, and eventually treating a gamut of neurological conditions including epilepsy, stroke, and autism. A variety of sensing mechanisms, such as functional-MRI, Electroencephalography (EEG), and Electrocorticography (ECoG), are commonly adopted to uncover patterns in both brain structure and function. In particular, the resting state fMRI (Kelly et al., 2008) has been proven effective in identifying diagnostic biomarkers for mental health conditions such as the Alzheimer disease (Chen et al., 2011) and autism (Plitt et al., 2015). At the core of these neuropathology studies is predictive models that map variations in brain functionality, obtained as time-series measurements in regions of interest, to clinical scores. For example, the Autism Brain Imaging Data Exchange (ABIDE) is a collaborative effort (Di Martino et al., 2014), which seeks to build a data-driven approach for autism diagnosis. Further, several published studies have reported that predictive models can reveal patterns in brain activity that act as effective biomarkers for classifying patients with mental illness (Plitt et al., 2015). Following current practice (Parisot et al., 2017), graphs are natural data structures to model the functional connectivity of human brain (e.g. fMRI), where nodes correspond to the different functional regions in the brain and edges represent the functional correlations between the regions. The problem of defining appropriate metrics to compare these graphs and thereby identify suitable biomarkers for autism severity has been of significant research interest. We show that the proposed two-sample test is highly effective at characterizing stratification based on demographics (e.g. age, gender) as well as autism severity states (normal vs abnormal) across a large population of brain networks.

**FIGURE 3:**
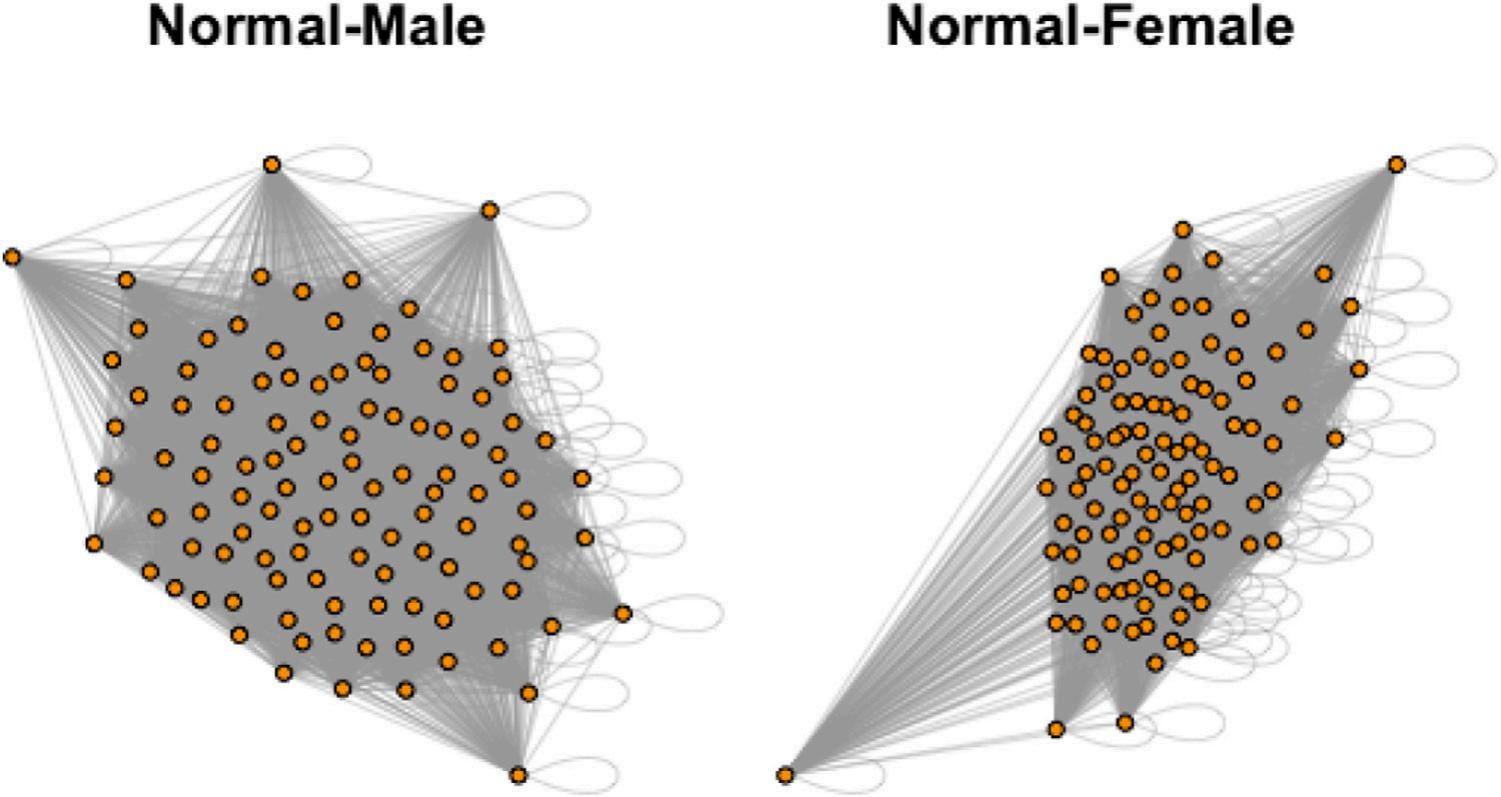
Example networks from Normal-Male and Normal-Female groups.

In the dataset, there are total 871 graphs and each graph consists of 111 nodes (functional regions). Through this example, we study the effectiveness of our approach under the weighted and undirected graph setting. In particular, we focus on detecting variations across stratification arising from demographics (gender, age). Specifically, groups of normal control subjects as well as those diagnosed with Autism Spectrum Disorders (ADS) are further sub-divided according to their gender (Male or Female) and age (under 20 or over 20), and we compare these sub-groups using the proposed test. Table 6 shows the distribution of graphs in the dataset and Figure 3 shows an example of the network structure of normal-male and normal-female groups.

**TABLE 6:** Distribution of graphs. “M” and “F” indicate male and female, respectively. ‘<20’ and ‘>20’ represent age less than 20 and over 20, respectively.
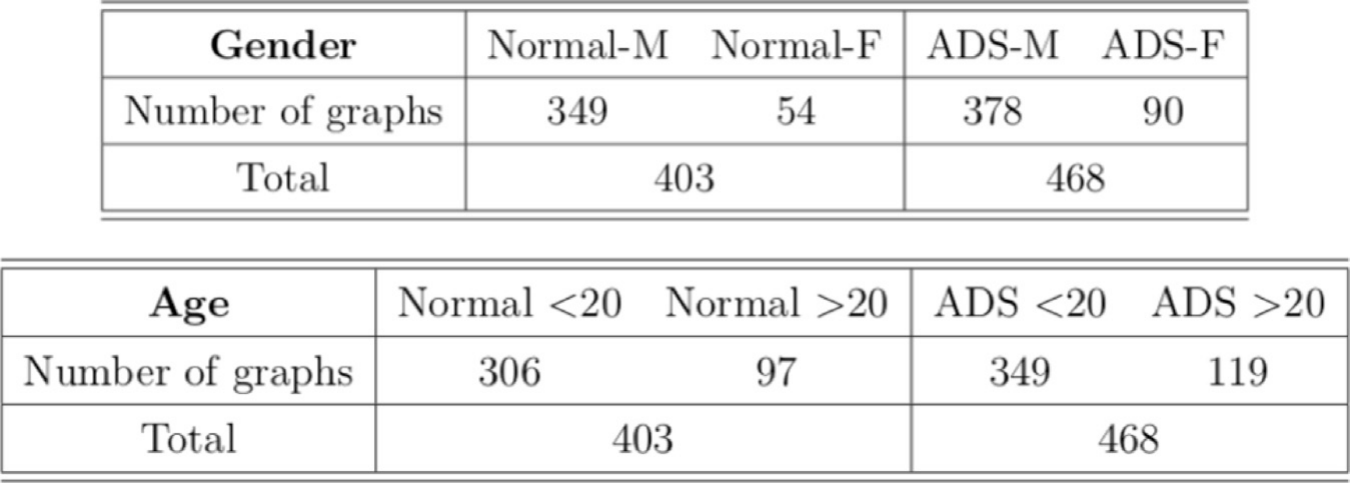

We conduct the two-sample test based on $T^{\prime }\_new$ for each group with 10,000 permutations and the results are summarized in Table 7. We see that the new test rejects the null hypothesis of homogeneity in groups with respect to the treatment and age at 5% significance level (Normal>20 vs ADS<20 and Normal<20 vs ADS>20). In addition, the new test rejects the null hypothesis of homogeneity in both normal and ADS groups with respect to the age difference (Normal<20 vs Normal>20 and ADS<20 vs ADS>20).

**TABLE 7:** *p*-values of the tests on the ABIDE dataset.
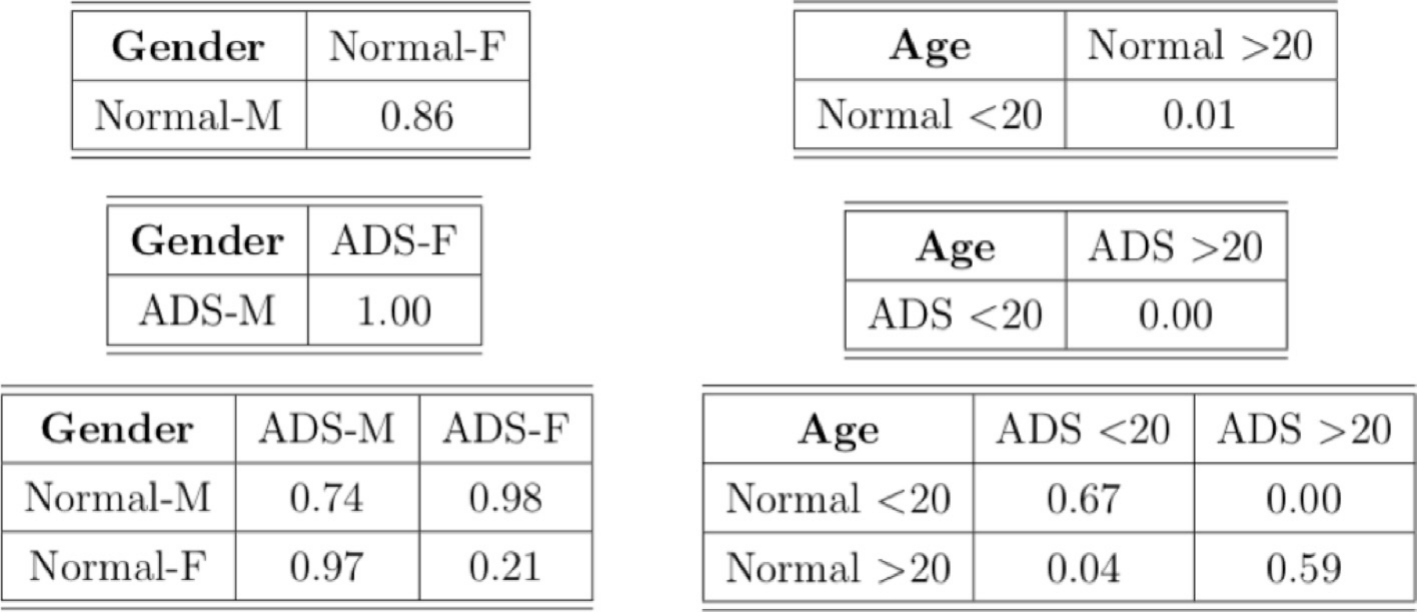

This conclusion indicates there is a dataset shift even within the same normal and ADS groups, depending on the age. Hence, the fact that normal and ADS groups are considered differently by age may affect the machine learning subjects classification and prediction task in population. Moreover, with the dataset in which the normal group and ADS group are determined differently by age and not by gender, the machine learning classification and prediction model may not be reliable. Hence, detecting dataset shift shed some light on the machine learning task for more reliable results.

We also compare the new test with the existing method T_fro to this example. Note that the existing method T_asymp may not be reliable due to the small number of nodes. Since T_fro is only applicable to the balanced sample sizes, we randomly choose 54 graphs from each group as the smallest sample size among the groups is 54. We run the tests 100 times at the significance level 5%. The test powers are shown in Table 8. We see that the new test in general outperforms T_fro. Compared to the results in Table 7, some examples show inconsistent performance of the tests. This is because we only consider a subset of graphs due to the limitation of the existing approaches in that they cannot be applied to unbalanced sample size examples.

**TABLE 8:** Estimated power of the tests with the significance level at 5%. Black numbers indicate the power of test based on T_fro and red numbers represent the power of test based on T'_new.
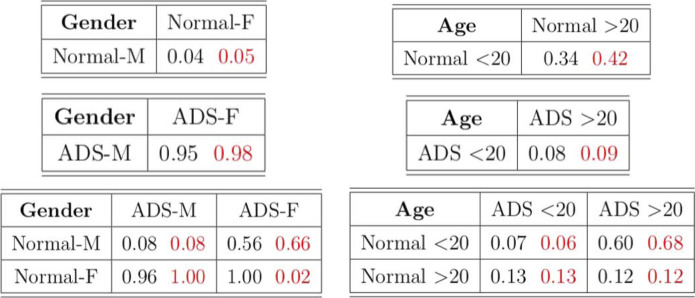

## 5 Conclusion

We propose the new two-sample test statistic for graph-structured data. Unlike the existing methods, the new test statistic is more versatile, which is applicable to directed graphs, imbalanced sample size cases, and even weighted graphs. The asymptotic distribution of the test statistic is presented and a practical testing procedure is proposed. The performance of the new method is studied under a number of settings. Experiments demonstrate that the new test in general outperforms state-of-the-art tests. The proposed test is also applied to two real datasets (including a safety-critical healthcare application), and we reveal that the new approach is effective to detecting the heterogeneity between disparate samples.

## Data Availability

The original contributions presented in the study are included in the article/Supplementary Material, further inquiries can be directed to the corresponding author.
